# Lack of stress responses to long-term effects of corticosterone in Caps2 knockout mice

**DOI:** 10.1038/srep08932

**Published:** 2015-03-10

**Authors:** Yuriko Mishima, Yo Shinoda, Tetsushi Sadakata, Masami Kojima, Shigeharu Wakana, Teiichi Furuichi

**Affiliations:** 1 Laboratory for Molecular Neurogenesis, RIKEN Brain Science Institute, Wako, Saitama, Japan; 2 CREST, Kawaguchi, Saitama, Japan; 3 Faculty of Science and Technology, Tokyo University of Science, Noda, Chiba, Japan; 4 Advanced Scientific Research Leaders Development Unit, Gunma University, Maebashi, Gunma, Japan; 5 Health Research Institute, National Institute of Advanced Science and Technology, Ikeda, Osaka, Japan; 6 RIKEN BioResource Center, Tsukuba, Ibaraki, Japan

## Abstract

Chronic stress is associated with anxiety and depressive disorders, and can cause weight gain. Ca^2+^-dependent activator protein for secretion 2 (CAPS2) is involved in insulin release. Caps2 knockout (KO) mice exhibit decreased body weight, reduced glucose-induced insulin release, and abnormal psychiatric behaviors. We chronically administered the stress hormone corticosterone (CORT), which induces anxiety/depressive-like behavior and normally increases plasma insulin levels, via the drinking water for 10 weeks, and we examined the stress response in KO mice. Chronic CORT exposure inhibited stress-induced serum CORT elevation in wild-type (WT) mice, but not in KO mice. Poor weight gain in CORT-treated animals was observed until week 6 in WT mice, but persisted for the entire duration of the experiment in KO mice, although there is no difference in drug*genotype interaction. Among KO mice, food consumption was unchanged, while water consumption was higher, over the duration of the experiment in CORT-treated animals, compared with untreated animals. Moreover, serum insulin and leptin levels were increased in CORT-treated WT mice, but not in KO mice. Lastly, both WT and KO mice displayed anxiety/depressive-like behavior after CORT administration. These results suggest that Caps2 KO mice have altered endocrine responses to CORT administration, while maintaining CORT-induced anxiety/depressive-like behavior.

Ca^2+^-dependent activator protein for secretion 2 (CAPS2) is a member of the CAPS/CADPS protein family, and was first identified as a protein associated with dense-core vesicle exocytosis in endocrine and neuroendocrine cells^1^,^2^,^3^. CAPS2 is involved in insulin secretion by mouse pancreatic β cells, as demonstrated by experiments showing that Caps2 knockout (KO) mice have reduced glucose-induced insulin secretion^4^. Additionally, CAPS2 appears to play important roles in the pathophysiology of mental disorders. Caps2 KO mice show abnormal psychiatric behaviors, including impaired social interaction and increased anxiety^5^,^6^.

Stress triggers the activation of the hypothalamic-pituitary-adrenal (HPA) axis^7^. Corticosterone (CORT), the major stress hormone produced in the cortex of the adrenal gland, plays a regulatory role in stress-induced HPA axis activity in rodents^8^. Chronically elevated CORT levels activate the chronic stress-response network, and as a result, impact various processes involved in coping with stress^9^. Previous studies showed that chronic CORT administration in rodents inhibits stress-induced serum CORT elevation^10^ and causes increases in serum insulin and leptin, adipose-derived hormones^11^,^12^,^13^ that regulate food intake and body weight^14^. In addition, chronic CORT administration normally induces depression in rodents^15^.

In the present study, we examined the relationships between CORT, insulin, obesity, and mental illness. Wild-type (WT) and Caps2 KO mice were treated with CORT via the drinking water for 10 weeks, and the effects of this chronic CORT intake were investigated. We suggest that Caps2 KO mice have an altered endocrine response to CORT, while maintaining CORT-induced anxiety/depressive-like behavior.

## Results

### Chronic CORT treatment does not suppress the increase in serum CORT levels after stress in Caps2 KO mice

**Figure 1:**
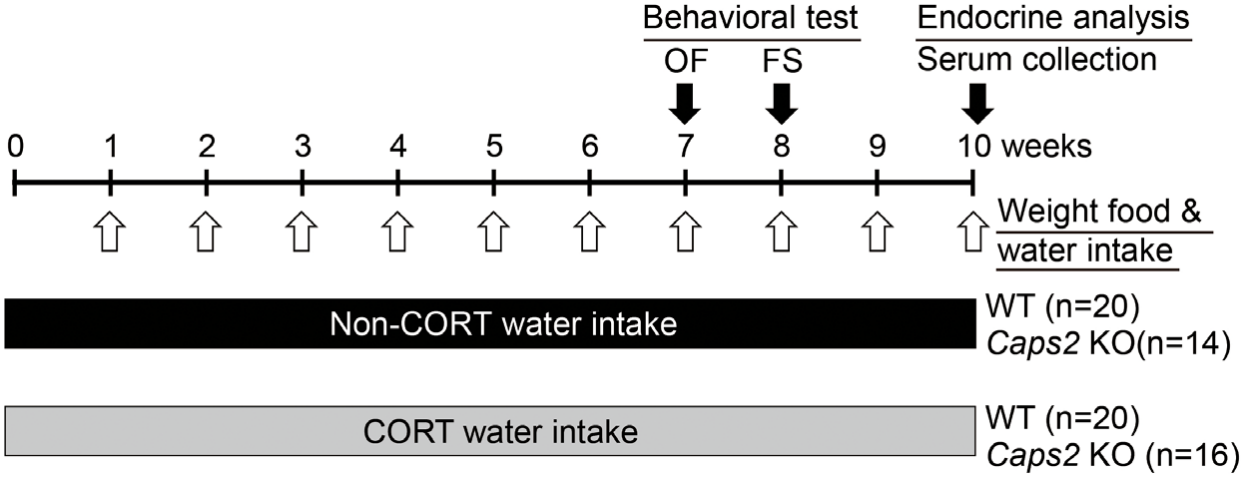
Experimental design. Both WT and Caps2 KO mice (male, 6–8-week-old) were given CORT-containing or non-CORT-containing water intake for 10 weeks (for number of mice for each group [n] and details, see Methods). Mice were subjected to behavioral analysis (open field test [OF] at 7 weeks and forced swim test [FS] at 8 weeks), and then sacrificed for endocrine analysis of their blood serum (at 10 weeks).

**Figure 2:**
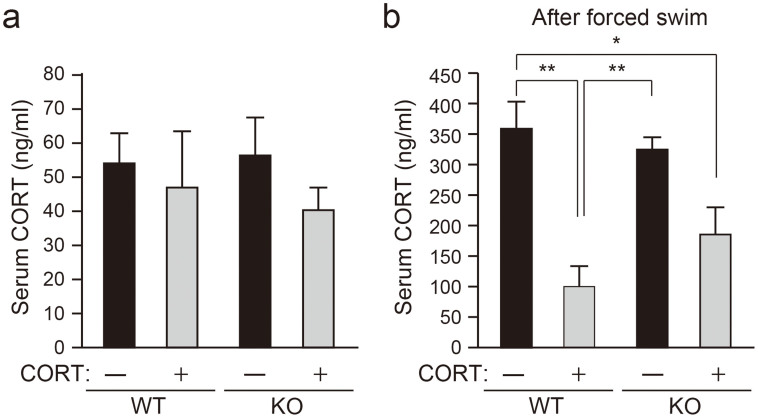
Swim stress increases serum CORT levels, but not in chronic CORT-treated WT mice. (a) Serum CORT was measured at the end of week 10 of treatment. Serum CORT levels were not significantly different among the groups. (b) Serum CORT was measured after the forced swim test on week 8 of treatment. Swim stress induced increases in serum CORT levels in the WT untreated group, but not in the WT CORT-treated group. In comparison, swim stress induced increases in serum CORT levels in both untreated and CORT-treated Caps2 KO groups. Values plotted are mean ± S.E.M (n = 8 per group). Two-way ANOVA (genotype and drug effect), followed by Tukey HSD: *P < 0.05, **P < 0.01.

To analyze the effects of the stress hormone CORT in Caps2 KO mice, male Caps2 KO (−/−) animals and their WT (+/+) littermates (6–8-week-old) were subjected to chronic CORT intake in their drinking water for 10 weeks (Figure 1). We measured circulating levels of serum CORT after treatment (Figure 2). There were no differences in basal CORT levels in the CORT-treated and untreated animals among WT and Casp2 KO mice in serum collected during the light phase (13:00–16:00) (Figure 2a). As reported in a previous study^10^, in WT mice, serum CORT levels after forced swim stress were increased in untreated animals, but was unchanged in CORT-treated mice (Figure 2b). However, this chronic CORT administration-induced suppression of stress-induced CORT elevation was insignificant in Caps2 KO mice (Figure 2b) (Tukey HSD *post hoc* test: WT untreated vs. CORT-treated, *P* < 0.0003; Caps2 KO untreated vs. CORT-treated, *P* < 0.12). These results suggest a differential response to stress between WT and Caps2 KO mice after chronic CORT treatment.

### Poor body weight gain in Caps2 KO mice is not related to food consumption during CORT treatment

**Figure 3:**
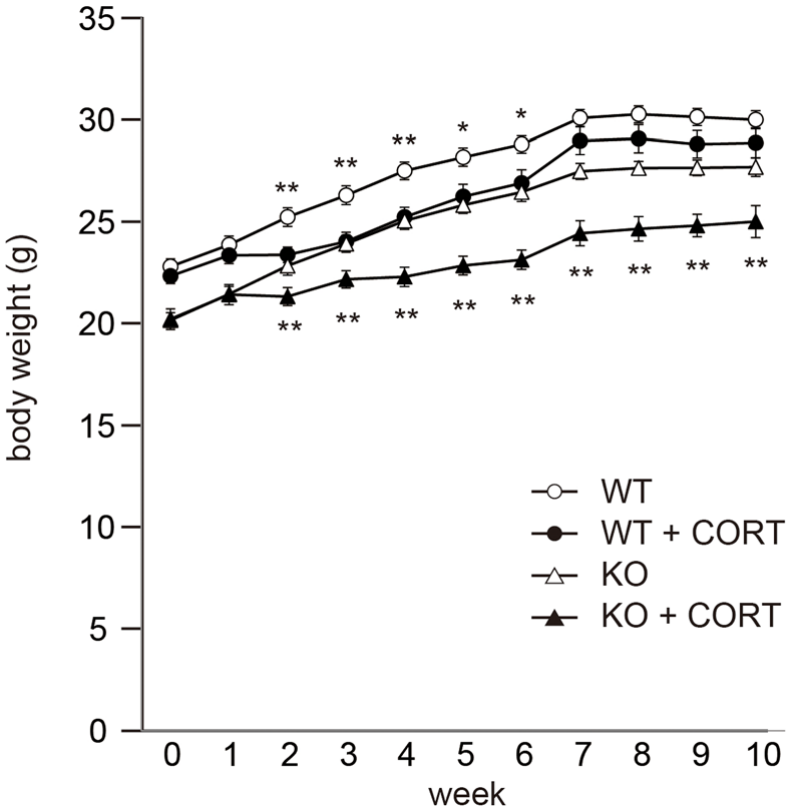
Chronic CORT administration causes a rapid decrease in body weight in Caps2 KO mice. Body weight was monitored during the 10 weeks of CORT treatment in both WT and Caps2 KO mice. Rapid body weight loss was observed in Caps2 KO mice. Values plotted are mean ± S.E.M. One-way ANOVA: *P < 0.05, **P < 0.01.

**Figure 4:**
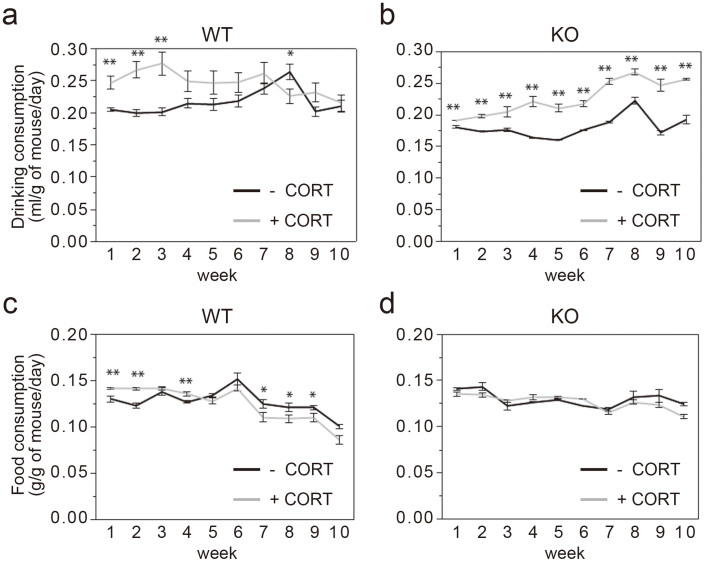
Chronic CORT administration affects dinking consumption but not food consumption in Caps2 KO mice. Drinking and food consumption were observed for 10 weeks in both WT (a, c) and Caps2 KO (b, d) mice. Values plotted are mean ± S.E.M. One-way ANOVA: *P < 0.05, **P < 0.01.

During the 10-week exposure to CORT, we monitored body weight, drinking and food consumption every week. Both WT and Caps2 KO mice treated with CORT showed poor weight gain, although there is no difference in drug*genotype interaction (Two-way repeated measures ANOVA: time*genotype [F = 2.56, *P* < 0.01]; time*drug [F = 14.64, *P* < 0.0001]; time*genotype*drug [F = 0.92, *P* < 0.52]). CORT-treated WT mice gained as much weight as untreated WT mice during the 7th week and beyond. In comparison, CORT-treated Caps2 mice displayed reduced weight gain during the duration of the experiment (One way ANOVA: *P < 0.05, **P < 0.01) (Figure 3). Compared with the respective untreated group, water consumption in CORT-treated WT mice was increased only during the first three weeks (Figure 4a), whereas it was increased in CORT-treated Caps2 KO mice over the entire period tested (Figure 4b). Compared with the respective untreated group, food consumption in CORT-treated animals was increased during weeks 1, 2 and 4, and reduced during weeks 7, 8 and 9 in WT mice (Figure 4c), whereas it was not changed in CORT-treated Caps2 KO mice (Figure 4d). These results suggest that chronic CORT exposure causes suppression of body weight gain in Caps2 KO mice, which is not due to a change in food consumption.

### Serum insulin and leptin levels in Caps2 KO mice are not increased by chronic CORT treatment

**Figure 5:**
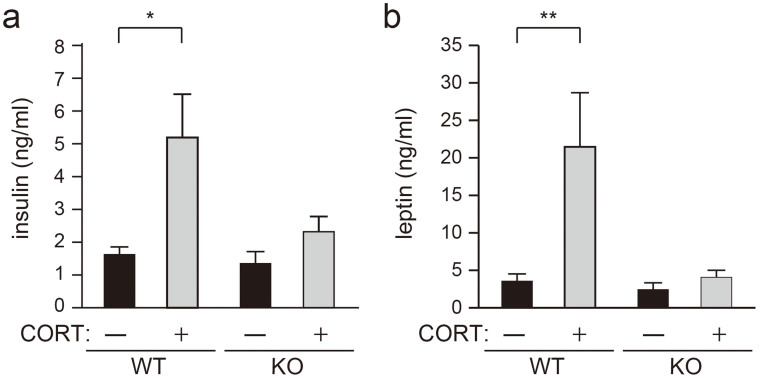
Defects in insulin and leptin responses to chronic CORT treatment in Caps2 KO mice. (a, b) Serum insulin (a) and leptin (b) levels were measured at the end of the 10th week of treatment. (a) Increases in serum insulin levels were observed in CORT-treated WT mice, but not in CORT-treated Caps2 KO mice. (b) Increases in leptin levels were observed in CORT-treated WT mice, but not in CORT-treated Caps2 KO mice. Values plotted are mean ± S.E.M (n = 8 per group). Two-way ANOVA (genotype and drug effect), followed by Tukey HSD: *P < 0.05, **P < 0.01.

Previous studies have shown that chronic CORT treatment causes increases in plasma insulin levels in mice^11^,^12^,^13^. We obtained serum during the light phase, and measured insulin levels by enzyme immunoassay (EIA). Similar to previous reports^11^,^12^,^13^, serum insulin levels were increased by chronic CORT treatment in WT mice (Figure 5a). However, serum insulin levels did not increase in Caps2 KO mice after chronic CORT treatment (Figure 5a) (Two-way ANOVA: genotype effect [F = 4.8399, *P* = 0.04]; drug effect [F = 10.69, *P* = 0.003]; interaction effect [F = 3.47, *P* = 0.07]. Tukey HSD post hoc test: WT untreated vs. WT CORT-treated, *P* < 0.007; Caps2 KO untreated vs. Caps2 KO CORT-treated, *P* < 0.74). We also measured leptin, a hormone made in adipose tissue, which is known to fluctuate according to insulin levels and body fat^14^. We obtained serum during the light phase, and measured hormone levels by EIA. Serum leptin levels were also increased by chronic CORT treatment in WT mice (Figure 5b). However, serum leptin levels were not increased by chronic CORT treatment in Caps2 KO mice (Figure 5b) (Two-way ANOVA: genotype effect [F = 5.6, *P* = 0.03]; drug effect [F = 6.45, *P* = 0.02]; interaction effect [F = 4.64, *P* = 0.04]; Tukey HSD post hoc test: WT untreated vs. WT CORT-treated, *P* < 0.02; Caps2 KO untreated vs. Caps2 KO CORT-treated, *P* < 0.99). These results suggest that serum insulin and leptin levels are not increased in Caps2 KO mice by chronic CORT treatment.

### Chronic CORT treatment in Caps2 KO mice causes anxiety/depressive-like behavior comparable to that in WT mice

**Figure 6:**
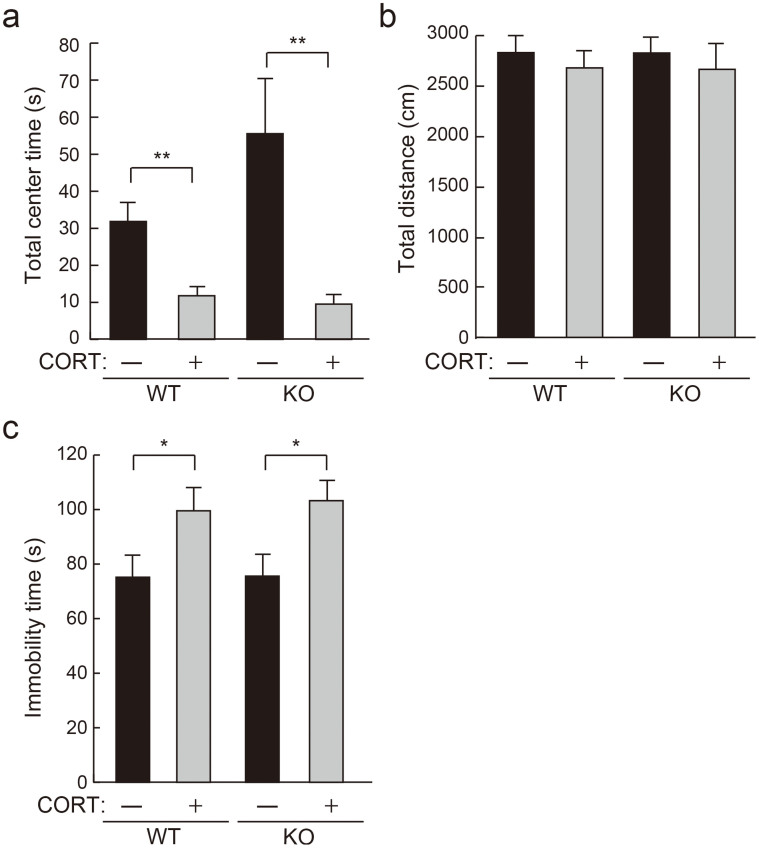
Chronic CORT administration causes a similar depressive and anxiety-like phenotype in both WT and Caps2 KO mice. The effects of chronic CORT administration on anxiety-like and depressive-like behaviors were analyzed with the open field test (a, b) at 7 weeks and the forced swim test (c) at 8 weeks, respectively. Results for CORT-treated (+) and untreated (−) groups among WT (left two bars; n = 20 and n = 20, respectively) and Caps2 KO (right two bars; n = 14 and n = 16, respectively) mice are shown. (a) Total center time shows time (s) spent in the center of the open field on day 3. Both CORT-treated WT and CORT-treated Caps2 KO mice showed a decrease in total center time. (b) Total distance (cm) traveled in the open field on day 3. There was no change in total distance traveled among the groups. (c) Total immobility time (s) in forced swimming. Total immobility time was increased in both CORT-treated WT and CORT-treated Caps2 KO mice. Error bars represent ± S.E.M. **P < 0.01, Mann-Whitney U test; *P < 0.05, Student's *t*-test.

Because chronic CORT treatment mimics anxiety/depression in rodents^15^, we tested CORT-treated mice for anxiety/depressive-like behaviors. To test for anxiety-related behaviors, we performed an open field test. Mice were tested for three consecutive days, and data from the third day was used for analysis. For total center time, which reflects anxiety, CORT-treated mice showed lower values compared to untreated mice among both WT and Caps2 KO mice (Figure 6a) (Mann-Whitney U-test: WT untreated vs. WT CORT-treated, *P* < 0.005; Caps2 KO untreated vs. Caps2 KO CORT-treated, *P* < 0.006). Analysis of total distance traveled showed that there was no difference among the groups, indicating that total locomotor activity was not affected by CORT treatment (Figure 6b). To test for depressive behaviors, we performed the forced swim test. Immobility time was increased in CORT-treated mice in both WT and Caps2 KO mice (Figure 6c), indicating that CORT treatment caused similar depressive-like behavior in both genotypes (Student's *t*-test: WT untreated vs. WT CORT-treated, *P* < 0.05; Caps2 KO untreated vs. Caps2 KO CORT-treated, *P* < 0.02). Moreover, in an elevated plus maze test, CORT-treated Caps2 KO mouse group showed a tendency to decrease time spent in open arm compared with the other groups (Figure S1), thereby suggesting increased anxiety in Caps2 KO mice after chronic CORT treatment.

## Discussion

In this study, both WT and Caps2 KO mice were treated chronically with CORT for 10 weeks. This chronic treatment did not impact basal serum CORT levels in either WT or Caps2 KO mice. However, serum CORT levels after swim stress were increased in untreated WT mice, but not in CORT-treated WT mice, similar to results reported in a previous study^10^. Because CORT is regulated by an inhibitory feedback loop, chronic CORT administration normally suppresses the HPA axis^7^. The swim stress-induced increase in CORT levels was not suppressed in CORT-treated Caps2 KO mice. Thus, in Caps2 KO mice, the CORT inhibitory loop may be impaired.

After week 6, body weight increased similarly in both CORT-treated WT and untreated WT mice. In comparison, CORT-treated Caps2 KO mice displayed less weight gain compared with untreated Caps2 KO mice although there is no difference in drug*genotype interaction. It is not yet clear why the difference in body weight changes between two genotypes occurred at later stage of chronic CORT treatment. Any metabolic changes after prolonged mild stress may be different between WT mice from KO mice with the loss-of-function of CAPS2-mediated insulin secretion. In addition, serum insulin and leptin levels were increased after CORT administration in WT mice as previously described^11^,^12^,^13^, but were unchanged in Caps2 KO mice. These results correspond with the roles of CORT and insulin in energy balance—CORT inhibits energy storage, causing a decrease in body weight, and insulin promotes energy storage, causing an increase in body weight^16^. Therefore, because insulin was secreted in response to CORT administration in WT mice, body weight increased similarly to that in untreated mice. However, because insulin was not increased in Caps2 KO mice, CORT administration caused poor weight gain in these animals.

We also conducted tests that evaluate anxiety/depressive behavior. Despite the defects in insulin and leptin release during CORT treatment, Caps2 KO mice did not exhibit heightened anxiety/depressive behavior compared with WT mice. This result suggests that chronic CORT administration does not exacerbate anxiety/depression in Caps2 KO mice.

Studies have shown that symptoms in adrenalectomized rats, which include reduced caloric intake and poor weight gain, are corrected by sucrose drink or intraventricular insulin and leptin administration^17^,^18^,^19^. These studies suggest that there is a metabolic feedback system that suppresses stress responses. However, the underlying regulatory mechanisms are unclear. CORT-treated Caps2 KO mice, which lack increased insulin release, may help to clarify the roles of metabolic feedback in stress responses.

Collectively, the findings of the present study show that chronic CORT treatment causes differential endocrine responses in Caps2 KO and WT mice. These differences in endocrine responses, however, were not associated with differences in anxiety/depressive behavior. This suggests that weight gain as a response to stress does not substantially impact anxiety-related stress. Most studies examining the roles of CORT, insulin and leptin in obesity and behavior have used adrenalectomized rats, which require complicated surgery. Thus, the CORT-treated Caps2 KO mice used in the present study may serve as a useful model for investigating the mechanisms of CORT and insulin in depression and obesity.

## Methods

### Animals

Caps2 KO mice^5^,^6^ and WT littermate controls (background: C57BL/6J) were used for the study. Male mice (6–8-week-old) were housed four per cage (according to genotype) and maintained under a 12:12 light-dark cycle (lights on at 08:00). The experiments were approved by the Institutional Animal Care and Use Committee of RIKEN and Tokyo University of Science. All experiments were conducted in accordance with the Regulations for Animal Research at the RIKEN and Tokyo University of Science.

### Drugs

CORT (Sigma, St. Louis, MO) was dissolved in ethanol (EtOH) and diluted in tap water at a concentration of 25 μg/ml for WT mice and 22 μg/ml for Caps2 KO mice (final EtOH concentration of 0.2% for WT mice and 0.18% for Caps2 KO mice). The concentration of CORT was adjusted according to the weight of each genotype. CORT or EtOH control was delivered in the drinking water, and solutions were replaced every 2–3 days.

### Experimental design

Both WT and Caps2 KO mice were divided into two groups; one group was treated with EtOH as a control (untreated group) and the other group was treated with CORT (CORT-treated group). Male mice were 7–8 weeks old at the start of the experiment. The experiment was repeated twice, and data were combined for behavioral testing. For the first set of mice, WT EtOH group, n = 12, WT CORT group, n = 12, Caps2 KO EtOH group, n = 8, Caps2 KO CORT group, n = 12. For the second set, WT EtOH group, n = 8, WT CORT group, n = 8, Caps2 KO EtOH group, n = 6, Caps2 KO CORT group, n = 6. Body weight, food consumption and drinking volume were measured once per week. After 7 weeks of CORT treatment, the open field test (days 48, 49, 50 or days 49, 50, 51) and the forced swim test (between days 55 and 59) were conducted. After 10 weeks, mice were sacrificed, and brain regions and serum were collected (Figure 1).

### Behavioral tests

Behavioral tests were performed between 13:00 and 17:00. Mice were acclimated to the behavioral room at least 1 h before testing.

#### Open field test

Mice were tested in the open field for 15 min per session for 3 consecutive days according to a previously published protocol^20^. The design permits assessment of exploratory behavior on days 1 and 2, and anxiety on day 3, because by day 3, mice are habituated to the open field. The open field test was performed as previously described^6^. Briefly, locomotor activity was measured with a light level of 70 lux in an open field apparatus (60 cm × 60 cm). Each mouse was placed at the left corner of the open field, and horizontal movements were recorded using Image J OF software (O'Hara & Co., Ltd., Tokyo, Japan). Total activity for 15 min on the third day was used for statistical analysis.

#### Forced swim test

The forced swim test was performed as previously described^21^. Briefly, mice were placed into plastic buckets, 19 cm in diameter and 23 cm deep, filled with water at 25°C, and mobility was recorded for 6 min. The last 5 min were scored for immobility. Immobility duration was scored using FST software (O'Hara & Co., Ltd.).

### Enzyme immunoassay of serum corticosterone, insulin and leptin

On week 8, blood was collected by tail incision after the forced swim test. On week 10, mice were decapitated, and trunk blood was collected during the light phase (13:00–16:00). Blood was collected in 1.5-ml plastic tubes and kept at 4°C overnight. Blood was centrifuged at 1,000 × g for 20 min, and serum was removed and stored at −80°C until use. Serum CORT was measured using an enzyme immunoassay kit (Assay Designs; Enzo Life Sciences, Farmingdale, NY). Serum insulin and leptin were measured using enzyme immunoassay kits (Millipore, Billerica, MA) (n = 6–8 for each group). Blood was also collected during the dark phase (20:00–22:00) on week 9 of CORT administration, and in the morning (09:00–11:00) on week 10, 3–4 hours before decapitation (data not shown).

### Statistical analysis

Data were analyzed using JMP software (SAS Institute, Cary, NC) and Excel (Microsoft, Redmond, WA). Data were analyzed by one-way, two-way or repeated measures ANOVA, followed by Tukey HSD post hoc test or Student's *t*-test. Values in graphs are expressed as mean ± SEM.

## Author Contributions

Conceived and designed the experiments: Y.M., Y.S., T.S., M.K., S.W. and T.F. Performed the experiments: Y.M. Analyzed the data: Y.M. and Y.S. Wrote the main manuscript text: Y.M. and T.F. Prepared figures: Y.M., Y.S. and T.F. All authors reviewed the manuscript.

## Supplementary Material

Supplementary Information Supplementary Information
