# Supplementary material for: Lack of stress responses to long-term effects of corticosterone in Caps2 knockout mice
Source: Sci Rep. 2015 Mar 10;5:8932. doi: 10.1038/srep08932 (PMC4354153; doi:10.1038/srep08932)
Supplement: Supplementary Information [file srep08932-s1.pdf]

Lack of stress responses to long-term effects of corticosterone in Caps2 knockout mice

Yuriko Mishima[1,2], Yo Shinoda[2,3], Tetsushi Sadakata[2,4], Masami Kojima[2,5], Shigeharu Wakana[6] and Teiichi Furuichi[1,2,3,*]

[1]Laboratory for Molecular Neurogenesis, RIKEN Brain Science Institute, Wako, Saitama, Japan; [2]CREST, Kawaguchi, Saitama, Japan; [3]Faculty of Science and Technology, Tokyo University of Science, Noda, Chiba, Japan; [4]Advanced Scientific Research Leaders Development Unit, Gunma University, Maebashi, Gunma, Japan; [5]National Institute of Advanced Industrial Science and Technology, Ikeda, Osaka, Japan; [6]RIKEN BioResource Center, Tsukuba, Ibaragi, Japan

*Corresponding author: tfuruichi@rs.tus.ac.jp

Supplementary Information

Figure S1. Chronic CORT treatment increased time spent in the closed arm of the elevated plus maze (EPM) in Caps2 KO mice compared with their WT littermates. The EPM test was performed as previously described (Sadakata et al., 2012). The EPM was set at a height of 65 cm and consisted of four grey Plexiglas® arms, each 8 cm wide × 25 cm long with 15-cm-high walls. Two arms were open, and two were closed. Male WT and Caps2 KO mice chronically treated with either 25 μg/μl CORT or 0.2% EtOH (vehicle) for 7 weeks were subjected to the EPM test. Mice in each of these four groups were individually placed in the centre of the maze, and the total distance and time spent in each arm (a, closed arm; b, open arm) were measured, and analysed with NIH Image Elevated Plus (E.P.) software (O'Hara & Co., Tokyo, Japan). (a) Total distance travelled (cm) during the last 3 min. (b) Total time spent in the closed arm (s). (c) Total time spent in the open arm (s). (d) Statistical data for the first 2 min and the last 3 min. There was no statistically significant difference in total time spent in each arm or in the total distance travelled among the experimental groups over the full 5-min period (data not shown) or during the first 2 min (d). However, during the last 3 min, there was a significant decrease in total time spent in the open arm in the CORT-treated Caps2 KO group compared with the other groups (c and d), although there were no significant differences in the horizontally travelled distance among the four groups (a). WT− (n = 6) and WT+ (n = 9) represent WT mice without and with CORT treatment, respectively. KO− (n = 6) and KO+ (n = 7) indicate Caps2 KO mice without and with CORT treatment, respectively. Error bars indicate SE. Two way ANOVA was used.

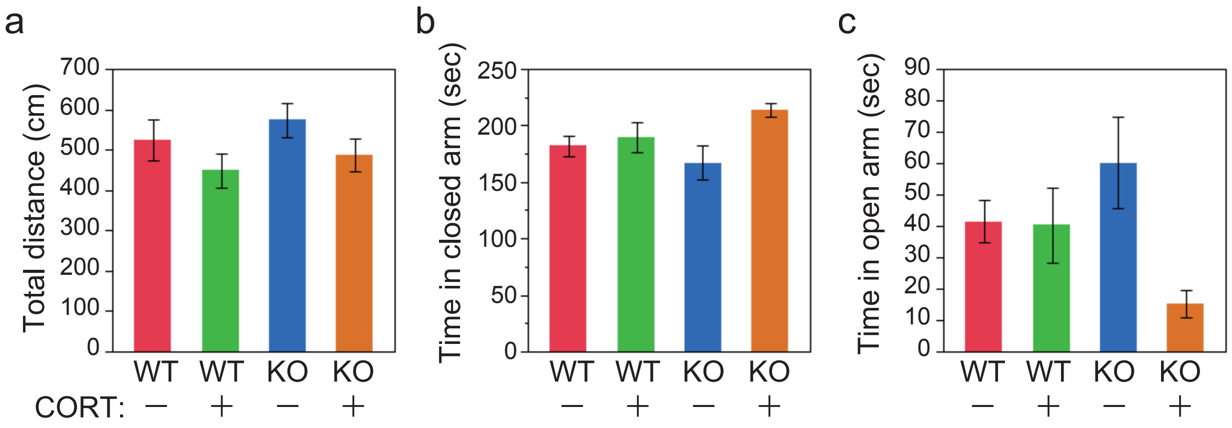

d

	P values	
Total time in closed arm	first 2 min	last 3 min
genotype	0.9308	0.6932
treatment	0.8231	0.0228*
genotype*treatment	0.5861	0.1045
Total time in open arm	first 2 min	last 3 min
genotype	0.8161	0.7586
treatment	0.7581	0.0294*
genotype*treatment	0.9541	0.0393*
